# A pre and post radiological image of Supracondylar fracture managed with open reduction and internal fixation

**DOI:** 10.11604/pamj.2022.41.319.29494

**Published:** 2022-04-20

**Authors:** Sakshi Pritam Arora, Waqar Mohsin Naqvi

**Affiliations:** 1 Department of Community Health Physiotherapy, Ravi Nair Physiotherapy College, Datta Meghe Institute of Medical Sciences, Wardha, Maharashtra, India,; 2 NKP Salve Institute of Medical Sciences and Research Centre, Nagpur, Maharashtra, India

**Keywords:** Supracondylar fracture, radiology, physiotherapy

## Image in medicine

A 46 years old female was dashed by a car from behind while crossing the road. The mechanism of injury followed a direct fall on the right elbow. She experienced sudden onset of severe pain followed by an inability to move the entire arm. As soon as she arrived in the hospital, she underwent a radiological diagnostic procedure which demonstrated Type-IV fracture (A) according to the Gartland classification of supracondylar fracture. The patient was showing complete restriction in elbow movement with pronation and supination of the forearm extremely painful. Hence, the patient then underwent open reduction and internal fixation with plates, screws, nails, and wire via lateral approach and was immobilized for six weeks. By the end of the immobilization period, she again went for the radiological diagnostic procedure to check the status of healing. As per the appearance of soft callus (B), she was prescribed physiotherapy treatment which mainly concentrated on pain, stiffness, restricted range of motion and reduced strength. The intervention involved passive mobility of each joint according to the indication of grades of mobilization, stretching of the agonist muscles and strengthening of the antagonist muscles followed by the progressive strengthening exercises. The physiotherapy was for six sessions, performed over a 5-week period beginning the week after cast removal. The functional independence was assessed using Functional Independence Measure (FIM) pre and post physiotherapy treatment suggesting modified independence by the end of the 12^th^ week of supracondylar fracture.

**Figure 1:**
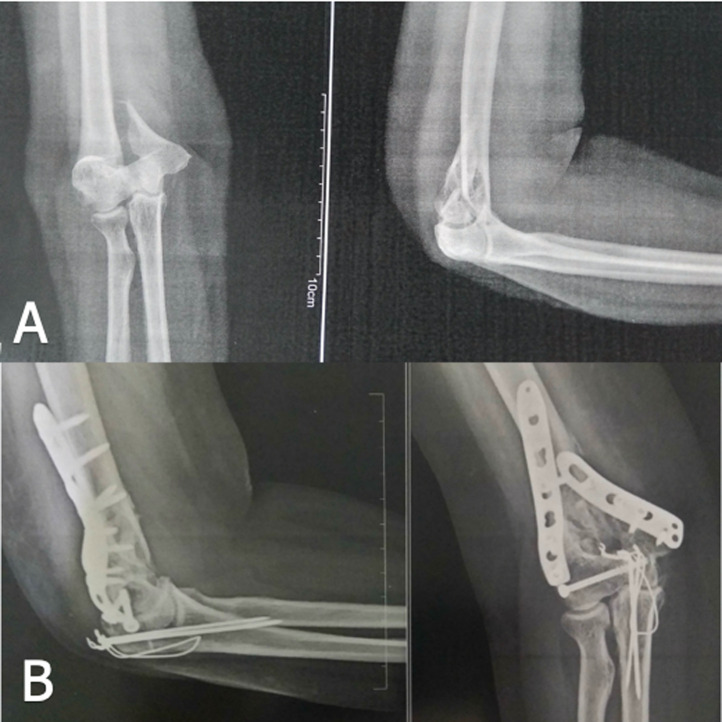
supracondylar fracture pre (A) and post (B) managed with open reduction and internal fixation with plates, screws, nail and wire

